# Hepatitis C virus infection in Irish drug users and prisoners – a scoping review

**DOI:** 10.1186/s12879-019-4218-6

**Published:** 2019-08-08

**Authors:** D. Crowley, R. Murtagh, W. Cullen, J. S. Lambert, T. McHugh, M. C. Van Hout

**Affiliations:** 1 Irish College of General Practitioners, Dublin, Ireland; 2 School of Medicine, University College, Dublin, Ireland; 3 Mater Misericordiae University Hospital, Dublin, Ireland; 4 Public Health Institute, Liverpool John Moores University, Liverpool, UK

**Keywords:** HCV, Prisoner, People who use drugs, PWUD, PWID, Irish, Scoping review

## Abstract

**Background:**

Hepatitis C infection is a major public health concern globally. In Ireland, like other European countries, people who use drugs (PWUD) and prisoners carry a larger HCV disease burden than the general population. Recent advances in HCV management have made HCV elimination across Europe a realistic goal. Engaging these two marginalised and underserved populations remains a challenge. The aim of this review was to map key findings and identify gaps in the literature (published and unpublished) on HCV infection in Irish PWUD and prisoners.

**Methods:**

A scoping review guided by the methodological framework set out by Levac and colleagues (based on previous work by Arksey & O’Malley).

**Results:**

A total of 58 studies were identified and divided into the following categories; Epidemiology, Guidelines and Policy, Treatment Outcomes, HCV-related Health Issues and qualitative research reporting on Patients’ and Health Providers’ Experiences. This review identified significantly higher rates of HCV infection among Irish prisoners and PWUD than the general population. There are high levels of undiagnosed and untreated HCV infection in both groups. There is poor engagement by Irish PWUD with HCV services and barriers have been identified. Prison hepatology nurse services have a positive impact on treatment uptake and outcomes. Identified gaps in the literature include; lack of accurate epidemiological data on incident infection, untreated chronic HCV infection particularly in PWUD living outside Dublin and those not engaged with OST.

**Conclusion:**

Ireland like other European countries has high levels of undiagnosed and untreated HCV infection. Collecting, synthesising and identifying gaps in the available literature is timely and will inform national HCV screening, treatment and prevention strategies.

**Electronic supplementary material:**

The online version of this article (10.1186/s12879-019-4218-6) contains supplementary material, which is available to authorized users.

## Background

Hepatitis C virus (HCV) infection is a major public health concern globally. In developed countries iatrogenic transmission of HCV has been substantially reduced and people who inject drugs (PWID) or have done so in the past are now the main group affected [1–5]. Of the 600,000 HCV infected people living in Europe an estimated 20,000–30,000 reside in Ireland [6–9]. An estimated 60% of HCV infections in Ireland remain undiagnosed [6, 10].

Ongoing criminalisation of drug users ensure high levels of HCV in prison populations globally. It is estimated that a quarter of the global prison population have been exposed to HCV infection [11–13].

Historically in Ireland, HCV transmission has occurred through infected blood and blood products with the majority of these infections occurring between the 1970s and the early 1990s [14]. Prior to the introduction of routine screening of Irish blood donations (1991), approximately 1700 people were infected through blood and blood products [6, 14]. HCV infection has been a notifiable disease in Ireland since 2004. Between 2004 and 2017, 14,107 cases were notified by the diagnosing laboratory with a peak in 2007 (*n* = 1539) [15]. In recent years there has been a decrease in cases notified with the number of new cases stabilising [15]. In 2012, the case definition was altered to specifically exclude resolved cases of HCV infection which may explain part of the reduction in the number of cases notified after this date [6]. Although there were no notifications of HCV infection prior to 2004, diagnostic data from the National Virus Reference Laboratory (NVRL) estimates that approximately 10,000 individuals were diagnosed with HCV infection between 1989 and 2004 [6]. A 2012 mathematical modelling study estimated the national prevalence of chronic hepatitis C in the Irish general population as 0.5–1.2% (20,000-50,000) [6]. A more recent lab-based residual sera study reported a prevalence estimate of 0.5-0.7% (20,000-30,000) [9]. Genotypes 1 and 3 are the types most commonly seen in Ireland and are distributed 55 and 45% respectively [16]. These are estimates of prevalence and the true prevalence rate in Ireland is unknown. There is no general screening of the population to determine prevalence rates with most studies only assessing the prevalence within specific risk groups [17, 18].

The estimates of prevalence however do indicate that in the general population the prevalence of hepatitis C is low, and most cases are from a defined risk group mainly PWID, people who received unscreened blood or blood products and people who were born in hepatitis C endemic countries [10].

Hepatitis C related morbidity and mortality continues to increase in Ireland, with increased hospital admission for, HCV related end stage liver disease (ESLD), hepatocellular cancer (HCC) and liver transplant [10]. This increase is likely related to the fact that the peak incidence of HCV infection in Irish PWID was in the late 1990s, and those infected during this period are now developing ESLD or HCC [19].

Since 2010 risk factor information has been available for 57% of notified chronic HCV cases in Ireland [6, 10, 14]. This data show injecting drug use (IDU) is the most common risk factor reported (80%), followed by possible sexual exposure (5%), receipt of blood or blood products (4%), vertical transmission (2%) and tattooing or body piercing (1%) (3). No risk factor was identified in 7% of notified cases [14].

The most significant risk factor for the transmission of HCV infection in Ireland is through injecting heroin. Capture-recapture studies carried out between 2001 and 2014 indicate that there are a significant number of problem opiate users in Ireland. The national point prevalence estimate of opiate users in 2014 was 18,988, giving a rate of 6.18 per thousand population aged 15–64 years (95% CI: 6.09–6.98). The prevalence of problem opiate use in Ireland has stabilised but remains amongst the highest in Europe [20]. Recent national trends indicate that the incidence of injecting opiates is declining [21]. There has been a steady decline in the total number of new entrants to treatment reporting opiates as their main problem drug with a drop from 58.1% in 2010 to 47% in 2016 [22]. The percentage who had ever injected among new treatment entrants for problem opiate use has also decreased significantly over time from 71% in 2004 to 64% in 2015 [21].

The Irish government introduced opiate substitution therapy (OST) and needle exchange programmes in Ireland in 1989 in response to an increasing HIV prevalence in PWID. OST is now provided nationally through a network of specialised HSE outpatient treatment clinics/satellite clinics, specially trained level 1 and 2 general practitioners in the community and prisons [23]. All OST patients are registered on a central treatment list (CTL). The number of clients on this list has increased each year since 2006 and there are now over 10,000 people on OST in Ireland [23].

The ongoing criminalisation of drug users in Ireland impacts on the levels of HCV infection in Irish prison populations. Over 60% of the 3400 prisoners incarcerated on a daily basis have a history of drug use with over a quarter reporting a history of IDU [13]. It is recognised that the majority of Irish prisoners come from marginalised communities with high levels of poverty, social deprivation, unemployment, illiteracy, physical and mental illness including addiction and blood borne virus (BBV) infections [24]. Like other prisoners, Irish inmates are identified as a hard to reach group for medical interventions and have poor access and uptake of traditional community medical services [24, 25].

Like other European countries, Ireland is upscaling HCV screening and treatment services [14, 26–28]. Direct acting anti-virals (DAA), mobile elastography and the movement of HCV treatment in the community, coupled with less restrictive HCV treatment guidelines have revolutionised the HCV treatment landscape [29–32]. Ireland’s national treatment program has signed up to the Global Health Sector Strategy on viral hepatitis for 2016–2020. The program aims to eliminate HCV as a major public health threat in Ireland by 2030 [33]. The roll out of new highly effective treatment in Ireland offers an exciting opportunity to achieve this goal yet many challenges to up scaling HCV screening and treatment to effective levels still remain. Understanding the burden of HCV disease and the barriers to screening, assessment, treatment and prevention in people who use drugs (PWUD) and prisoners in an Irish context is imperative in the planning and implementation of an effective national HCV strategy.

This scoping review collects and summarises the literature on HCV infection in PWUD and prisoners residing in Ireland. This review can, act as a baseline by which to monitor progress, support focused action and inform national and international public health HCV elimination strategies that target PWUD and prisoners as key at risk populations. This review also aims to identify gaps in the Irish-based HCV literature related to PWUD and prisoners and assess how these impacts on strategy development and service delivery.

## Methods

This scoping review of HCV infection in drug users and prisoners in Ireland was conducted by applying the methodological framework set out by Levac and colleagues which was based on prior work by Arksey & O’Malley [34, 35]. The steps include; identifying the research question, identifying all relevant studies, selecting significant studies, charting the relevant data and then summarising and reporting the results. The rationale for using this methodology was to determine what is known about HCV infection in Irish PWUD and prisoners by reviewing all available literature (quantitative, qualitative, guidelines and policy documents) on these two groups and to map the key findings and concepts emerging from this body of literature. A further aim was to map gaps in the literature which will inform future research.

A broad search strategy was conducted in order to identify all relevant studies. Databases searched included PubMed, Science Direct, EMBASE, PsycINFO, Cochrane library and Medline. No limits were placed on dates. The key terms we used for this search were the MeSH terms, “hepatitis C” and [“prison” or “prisoner”] and “drug users” and “Ireland” or similar non-MeSH terms outside PubMed (Additional file 1). Follow up search strategies included hand searching relevant national websites including the Health Service Executive (HSE), Irish Prison Service (IPS), Departments of Health and Justice (DOH and DJ), the Irish Penal Reform Trust (IPRT), Health Surveillance Protection Centre (HSPC) and European Monitoring Centre for Drugs and Drug Addiction (EMCDDA). National experts and authors of existing papers were contacted to identify possible sources of unpublished and grey literature. Reference list were also manually searched by the team to identify relevant studies or literature not captured. References were managed by the citation manager Endnote®. This software facilitated the recording and organisation of all relevant literature and also allowed for the cross checking of data records, removal of duplicates and extraction of information from the papers included in the review. The literature search was completed in June 2018.

Eligibility criteria for study selection centred on whether studies broadly reported on any aspect of HCV infection in Irish PWUD or prisoners including those that reported findings from analysis of these groups within broader populations. Publications to be included were peer-reviewed research, published reports, guidelines and strategies, editorials, commentaries and audits. Articles were excluded if they did not report results on these specific cohorts. Researcher 1(DC) analysed all articles found by title to select those that were potentially relevant. The abstracts of all studies selected based on their titles were independently evaluated by researcher 2(RM) and any discrepancies were kept in the analysis.

Data was extracted and charted from all studies selected at the abstract stage, using an instrument designed for this study covering; date and author, setting, study population and sample size, data collection period, study design, HCV prevalence and associated risk factors, HCV incidence, treatment outcomes, barriers and facilitators to HCV care, knowledge and experience of living with HCV, recommendations on the management of HCV and other outcomes. Data was extracted for each paper by a single researcher (DC) and checked by a second researcher (RM) to ensure that data extraction was accurate and comprehensive. The final decision to include studies was made based on this data extraction and whether it met the inclusion/exclusion criteria, based on independent evaluation by two authors (DC and RM), and a discussion of any discrepancies with the third author (TMcH).

A qualitative synthesis of the literature was carried out. The process of navigating and redefining the findings was iterative, and the researchers engaged with each stage in a reflexive manner, by fine tuning and repeating steps so as to ensure comprehensive synthesis of literature. Papers reporting on HCV incidence, prevalence, risk factors and screening uptake were categorised as epidemiological and reported and interpreted according to year of publication. For ease of reporting and interpretation this largest category was sub-categorised into those published in the last decade (2008–2018) and more historical studies published before 2008. The identified studies in this category reported on different populations (prisoners, PWID and PWUD on OST) and settings (prison, drug treatment centres and general practice).

The majority of the relevant literature retrieved from the web-site searches were either national reports, strategy documents or guidelines and were categorised as such. Studies reporting on outcomes following HCV diagnosis (self –clearance and sustained virologic response (SVR)) were categorised as treatment outcomes. The remaining literature was a mix of qualitative and quantitative studies that reported on a variety of outcomes including HCV –related health issues, alcohol consumption and patient and health provider experience of HCV infection and/or treatment and for convenience of reporting were categorised into HCV-related health issues and patient and health- providers’ experience. Where studies reported on a number of outcomes that crossed categories, these studies are categorised in the tables according to main/primary outcome and each outcome is reported in the result section according to its appropriate category.

## Results

**Fig. 1:**
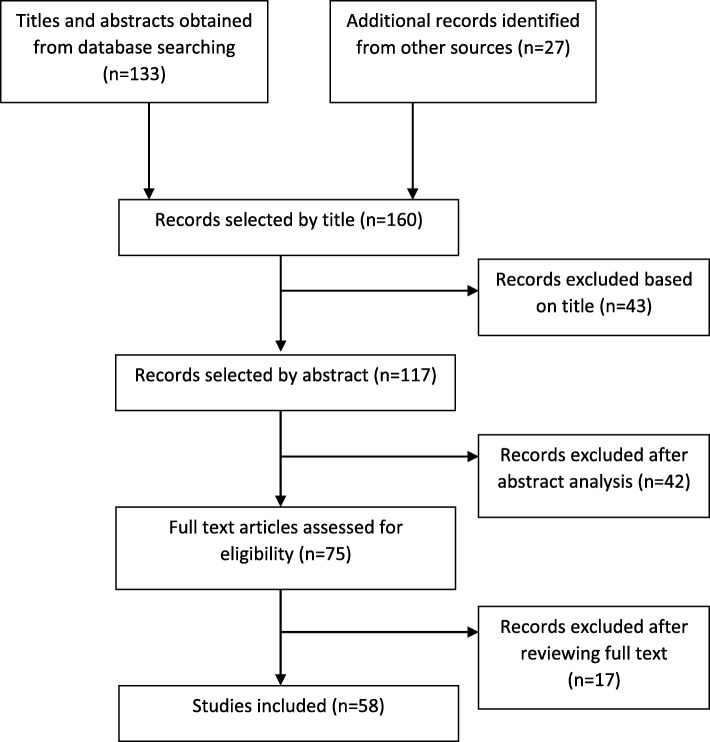
Flow diagram

Initial screening identified a total of 160 of which 27 were from grey literature and manual searching of reference lists. 117 met the inclusion criteria on title. Following a review of abstracts 42 studies were excluded. The remaining 75 studies were read in full and a further 17 were excluded. A total of 58 studies covering the period 1983–2018, were included in the final data synthesis (Fig. 1).

Five broad categories were identified to assist in the organisation of the literature. These were; *Epidemiology*, *Guidelines and Policy*, *Treatment Outcomes*, *HCV -related Health Issues* and qualitative research reporting on *Patients’ and Health Providers’ Experiences.*

## Epidemiology (32)

For clarity the epidemiological studies have been grouped into studies reporting on HCV incidence and prevalence pre and post 2008. Within these time frames the studies are further categorised according to study population (prisoner, PWID and PWUD) and community location (drug treatment centre or primary care). The findings from studies reporting on risk factors and HCV screening uptake along with identified gaps in surveillance data are reported at the end of this section.

### Pre-2008 (22 studies)

#### Incidence (2 studies)

Two studies reported on HCV incidence in the pre-2008 period. The first was a 2003 a single-site study on HCV negative PWID attending methadone maintenance treatment (MMT) at the National Drug Treatment Centre (NDTC). Follow up screening on 100 patients found an HCV incidence of 66 (CI 51–84) per 100-person years (py). A larger 2005 study involving 21 drug treatment centre sites and 1459 PWUD reported an incidence of 24.5 per 100 py. [36, 37].

#### Prevalence (20 studies) (prisoners =3 studies, PWUD = 7 studies, PWID = 8 studies)

A total of papers was identified reporting on the prevalence of HCV infection in PWUD and prisoners in Ireland dating from 1983 to 2008.

### Prisoners

Two studies were prison-based, one a randomised national study of the general prison population, the other was conducted on committal prisoners only. The 2000 study of 9/15 prisons in the Republic of Ireland (ROI) reported an anti- HCV prevalence of 37%, increasing to 81% among those prisoners with a history of IDU. The 2001 multi-site committal study reported an anti- HCV prevalence of 3% in those who had never been imprisoned previously, 22% in prisoners aged 19–25, increasing to 72% in prisoners with a history of IDU [38].

An interesting 2000 prison-based study reported on the discrepancy between self-reported HCV status and actual status (confirmed by oral swabs). Based on self-report the HCV prevalence estimates were 19% but actual prevalence was double this rate (37%) with small numbers (5%) reporting being anti-HCV positive erroneously [16].

$$ PWUD\left(\mathrm{Drug}\ \mathrm{Treatment}\ \mathrm{Centres}=4\ \mathrm{studies},\mathrm{Primary}\ \mathrm{Care}=3\ \mathrm{studies}\right). $$

Nine studies reported on HCV prevalence in PWUD attending MMT in community drug treatment clinics. The first was a 2001 multi-site randomised chart review of 715 patients attending MMT. This study reports an anti-HCV prevalence of 78.8% which reduced to 52% in those under 25 [39]. The 2005 incidence study described above reported an HCV prevalence of 66% in a population of PWUD attending drug treatment across 21 sites in the Dublin region [37]. A single site study from the NDTC reports an anti-HCV prevalence of 81.6% among study participants attending the centre for MMT during a 3-month period in 2002 [40]. A unique 2004 study reported an anti-HCV prevalence of 27% among adolescent drug users attending drug treatment, increasing to 55% among those with a history IDU [41].

A 2003 study involving 531 PWUD attending MMT at 42 general practice locations reported an anti-HCV prevalence of 78% [42]. A single site 2005 study reported an anti-HCV prevalence of 88% [43]. A later 2007 multi-site study reported an anti-HCV prevalence of 69% [44].

$$ PWID\ \left(\mathrm{Drug}\ \mathrm{Treatment}\ \mathrm{Centres}=7\ \mathrm{studies},\mathrm{Primary}\ \mathrm{Care}=1\ \mathrm{study}\right). $$

A 1983 paper reported on the identification of non-A non-B exposure in 90% of a cohort of PWID with acute hepatitis undergoing biopsy at a single hospital site in Dublin [45].

Four studies were single site studies from the NDTC (1995–2000) three of which were on new entrants with varying lengths of IDU history. Among new entrants HCV prevalence ranged from 52 to 61.8% [17, 46, 47]. The single 1995 study reporting on HCV prevalence among PWID on methadone maintenance treatment (MMT) reported an anti-HCV prevalence of 84% [48].

A 2003 single-site study was the first to report on the prevalence of chronic HCV infection and genotype distribution in a PWID cohort. Of the 94 included in the prevalence part of this study, 74.5% were anti-HCV positive, 41.5% where HCV-RNA positive (chronic HCV infection). The genotype distribution was 66.6% genotype 1 and 25.6% genotype 3. This study also included a mathematical modelling component to estimate national HCV burden levels [49]. These findings are reported in a later section of this review. A multi-site 2005 study involving 496 patient’s mono-infected with HCV of whom 127 (with demonstrated self- clearance on initial testing) were followed up 2 years later. The initial testing showed a self-clearance of 38% which when followed up 2 years later had reduced to 31.1%. The genotype distribution reported in this study was genotype 1 = 48.8% and genotype 3 = 48.5% [50]. A 2005 multi –site study (10 sites including 2 residential drug treatment sites) reported an anti-HCV prevalence of 61% among PWID who had a history of IDU in the past 6 months but who had never tested for HCV [51].

Only one single-site study reported specifically on HCV infection in PWID in primary care. This unique longitudinal study followed a cohort of 82 PWID attending a south inner-city Dublin general practice over 25 years. At the beginning of the study HCV infection had not been identified but at 10-year follow-up, 33% of those still alive had been infected. At 25-year follow-up 40% of survivors were anti-HCV positive [52].

### *At -risk population associated with PWUD* (2 studies)

Two studies reported on HCV prevalence in groups associated with PWUD. A single 2001 study reported that 82% of infants referred to an outpatient paediatric HCV clinic where from mothers with a history of IDU [53]. A 2008 study of homeless people found high levels of PWUD among this group (64%) as well as a high prevalence of HCV exposure (36%) [54] (Table 1).

**Table 1:** Epidemiology Pre-2008

	Date and author	Setting	Sample (n)	Data collection	Design	Main results
Prisoners						
[18]	2000 Alwright et al	National Multi-site 9/15 prison locations	Prisoners (1205; m = 1148)	Sept – Nov 1998	Cross-sectional randomised prevalence study	Anti-HCV = 37% (95% CI = 34.3 to 39.9%)
						• 80% among prisoners with a Hx of IDU
						• 60% of women and 42% of men had Hx of IDU
						• 20% first IV in prison and 71% shared needles
						Significant risk factors (p < 0.05): • IDU • Hx of sharing while in prison
[16]	2000 Thornton et al	National Multi-site 9/15 prison locations	Prisoners (1205; m = 1148)	Sept – Nov 1998	Cross-sectional study comparing reported vs actual HCV status	Anti-HCV = 37%
						Self-report anti-HCV = 19%
						• Among those self-reporting being anti-HCV positive, 5% were negative on oral fluid assay.
						• Among those reporting a previous negative result, 37% were positive on oral fluid assay.
[38]	2001 Long et al	National Multi-site 5 of 7 committal prisons	Prisoners (607; m = 555)	April–May 1999	Cross sectional prevalence study on new entrants and risk factors	Anti-HCV = 22% (CI: 19–25%)
						• anti HCV = 72% (Hx of IDU)
						• anti HCV = 3% (Never in prison)
						Significant risk factors (p < 0.05): • IDU
PWUD						
[39]	2001 Fitzgerald	Dublin Multi-site 5 Drug Treatment centre sites	Opiate users on MMT > 4 weeks (138/715; m = 99)	1997	Cross-sectional retrospective prevalence study - randomised sample (chart review)	Anti-HCV = 78.8% • 60% screened • Among those < 25 yrs., anti-HCV prevalence decreases to 52%
[42]	2003 Cullen et al	ERHA Multi-site 42 GP locations	Drug users on MMT (531; m = 443)	Not reported	Cross sectional prevalence study and associated risks	Anti-HCV = 78%
						• 67% screening documented • 193 had screen completed by GP, 74 another service and 113 no screen but self-report from patient
						• Predictors of being screened
						• Hx of imprisonment
						• documented HIV neg • Hx of IDU
						Significant risk factors (p < 0.05):
						• age > 26
						• Hx of IDU
						• Hx of imprisonment drug use prior to 1989
						• HIV pos/HBV pos
[41]	2004 Moloney et al	Dublin Single-site Community drug treatment programme for young people	Adolescent drug users (54; heroin smoking = 64%)	1998–2001	(Letter – response to Kavanagh et al. 2003)	Anti-HCV = 27% Anti-HCV among declared injectors = 55% Mean duration of injecting:
						• Anti-HCV positive = 1.42 yrs.
						• Anti-HCV negative = 1.16 yrs.
[37]	2005 Grogan et al	Dublin Multi-site Community drug treatment centres (21)	Drug users on MMT (358; m = 214)	2001	Cross-sectional- retrospective (one in four consecutive sampling)	Anti-HCV = 66%
						Incidence 24,5 per 100 years
						88% tested for HCV
						Significant risk factors (p < 0.05):
						• age > 25
[44]	2007 Cullen	Multi-site 25 GP practices Dublin	PWUD on MMT (196; m = 100)		Cross-sectional Prevalence	Anti-HCV = 69%
						• 77% screened for HCV
						• 36% of those anti-HCV were tested for HCV-RNA
						• 30% were referred to hepatology
						• 24% attended the clinic
						• 13% had a liver biopsy
						• 3% had started treatment
[54]	2008 O’Carroll et al.	DublinMulti-site	Homeless (393; m = 61%; drug users = 64%)	2005	A census of homeless adults (researcher administered questionnaire)	Anti-HCV = 36% (95% CI: 31–41%)
[40]	Noonan et al. 2009	Single site NDTC	Drug users receiving MMT (103; m = 67)	Sept-Dec 2002	Cross-sectional survey	Anti-HCV = 81.6% (untested = 2.8%)
						HCV RNA = 15.6% (untested = 65%)
						• Most of the study participants had accurate self-reported of HCV status
						Problem drinking:
						• Prevalence (audit score) = 41% (95% CI 33–51%).
						• 98% agreed that ‘alcohol may worsen HCV related liver disease’ • 92% agreed that ‘reducing
						• alcohol consumption may help HCV related liver disease’.
**PWID**						
[45]	1983 Fielding et al.	Dublin Single site Hospital	PWID with acute hepatitis undergoing biopsy (27; m = 22; mean age = 20.8 yrs.;Hx IDU = 6–120 months)	Jan-April 1981	Cross-sectional prevalence study	90% had exposure to non-A non-B (HCV)
[48]	1995 Smyth et al.	Dublin Single site Specialist drug treatment service (NDTC)	PWID on OST (272; m = 194)	Aug 1992–1993	Cross-sectional prevalence study	Anti-HCV = 84%
						Significant risk factors (p < 0.05):
						• male gender
						• > 2 years Hx IDU
[17]	1998 Smyth et al.	Dublin Single site Specialist drug treatment service (NDTC)	PWID - new entrants to treatment (733; m = 529)	1992–1997	Cross-sectional prevalence study	Anti-HCV = 61.8%
						(95% CI = 58.3–65.3) Significant risk factors (*p* < 0.05):
						• older age
						• longer HX of IDU
						• IDU before 1990
						• daily expenditure > 65 punts
[47]	1999 Smyth et al	Dublin Single site Specialist drug treatment Service (NDTC)	PWID - new entrants with HX IDU of < 25 months (353; m = 241)	July 1993- Dec 1996	Cross-sectional prevalence study	Anti-HCV = 52.1% • ↓ risk for those starting IDU after 1993 and with IDU < 13 months
[55]	2000 Smyth et al	Dublin Single site Specialist drug treatment service (NDTC)	PWID, first time attendees (119; m = 84)	July 1996–January 1997	Prospective evaluation of an HCV testing algorithm	Anti-HCV = 54% • 21/119 completed assessment
						• 48 tested for HCV
						• 13/26 HCV infected left OST treatment before receiving result
						• 4/19 attended on-site hepatology services
[53]	2000 Healy et al	Dublin Single site Hospital (Infectious paediatric OPD)	HCV+ mothers of infants referred to Paediatric infectious disease service (296; PWID = 244)	1994–1999	Cross-sectional Prevalence	HCV RNA: 55% (84 tested) • 82% infected via IVDU
[49]	2003 Kavanagh et al	Dublin Single-site	PWID (94 - prevalence study; m = 63) PWID = 5519 (mathematical modelling)	November 2001	Prevalence of HCV and its Prognostics/co-factors Mathematical modelling to estimate national HCV burden	Anti-HCV = 74.5% HCV RNA = 41.5% • Genotype 1 = 66.6%
						• Genotype 2 = 2.6%
						• Genotype 3 = 25.6%
						Modelling predictions:
						• HCV cirrhosis = 1214 cases (20 yrs.)
						• HCC = 35 per annum
						• Hepatic decompensation = 60 per annum
						• Liver related deaths = 50 per annum
[36]	2003 Smyth et al	Dublin Single site Specialist drug treatment service (NDTC)	HCV negative PWID (313; Follow up repeat HCV test = 100)	Nov 1992 – September 1998	Retrospective cohort study Incidence of HCV infection	HCV seroconversion = 67% Incidence = 66 per 100-person years (CI: 51–84 per 100-person years)
						Of 74 patients who were retested within 24 months
						• HCV seroconversion = 61% • Incidence = 100 infections/100
						• person years (95% CI: 73/100 to 134/100 person years).
						Significant risk factors (p < 0.05): • Hx IDU
						• Hx imprisonment
[51]	2005 Smyth et al	Dublin Multi-site (10) Community drug treatment clinics (7) Residential drug treatment centres (2) NDTC (1)	PWID - IV in the past 6 months / not tested for HCV (159)		Cross-sectional survey	Anti-HCV = 61%
						Predictors of positive test result (p < 0.05): • increased total number of lifetime injecting episodes
						• closer social relationships with other IDUs
						• injecting in the home of other IDUs
						Non-predictor of positive result (*p* > 0.05): • Frequency of recipient syringe sharing
						• backloading
						• sharing of injecting paraphernalia
[56]	2006Cullen	Dublin Multi-site 26 GP practices	PWID-GP	2005 (6-month period)	Cluster randomised trial /evaluating the effects of HCV guidelines	Intervention group • ↑ HCV screening (OR = 3.76; 95% CI = 1.3 to 11.3)
						• ↑ referral to a hepatology clinic (*p* = 0.06)
[6]	2012 Thornton et al	National	General population (6387; m = 4024)	1989–2009	National HCV prevalence study using data collected from NVRL (1989–2004) notification data (2004–2009) Mathematical modelling	General population estimate for chronic HCV infection = 20,000–50,000 (0.5–1.2%). • 10,000 people diagnosed anti-HCV between 1989 and 2004, peaking in 2000.
						• Genotype 1 = 55% and Genotype 3 = 39%.
						• Drug use most likely risk (80%).
						• Median age of diagnosis is 28. • 70% of those infected though drug users were male. • Median age at diagnosis for those infected through drug use = 25 years for males and 23 years for females.

### Post 2008 (10 studies)

#### Incidence (1 study)

A 2016 study using mathematical modelling and data on 14,320 PWID registered on the NDTRS estimated that 12,423 (CI 10,799-13,161) PWID were infected with HCV from 1991 to 2014, 9317 (CI 8022-9996) of whom had chronic infection. This paper also reported that new infections peaked in 1997, and that almost three quarters of those infected have the disease for greater than 10 years [19].

### Prevalence (9 studies) (Prisoner = 2 studies, PWUD = 6 studies, PWID = 1 study)

#### Prisoner

A 2014 study conducted in 13/15 of the prisons in the ROI reported an anti-HCV prevalence of 13% (95% CI 10.9–15.2%) among the general prison population, increasing to 41.5% in prisoners with a history of IDU and 54% in those with a history of injecting heroin [13]. The most recent prison study from 2014 (single –site) reported an HCV prevalence of 37% among prisoners on MMT [57].

$$ PWUD\left(\mathrm{Drug}\ \mathrm{Treatment}\ \mathrm{Centres}=5\ \mathrm{studies},\mathrm{Primary}\ \mathrm{Care}=1\ \mathrm{study}\right). $$

Two 2014 studies identified from the grey literature reported on HCV infection in PWUD attending MMT in drug clinics outside of Dublin and reported an anti- HCV prevalence less than Dublin based studies (24%%) [58] (Horan A: Chart audit of HCV screening measuring the effect of chart labelling, unpublished). A published 2017 study reported an anti-HCV of 63.6% among PWUD attending MMT at a North Dublin inner city treatment centre [59]. Two recent large HCV screening audits, identified through the grey literature search, reported an anti-HCV prevalence of almost 80% and a chronic HCV prevalence of 65% among PWUD attending MMT at 23 drug treatment clinics in Dublin (Burke M: Audit of HCV screening using retrospective patient records, unpublished), (Burke M. Audit of HCV screening using retrospective patient records [Unpublished audit]). The most recent prevalence study in PWUD attending OST in general practice reported an anti-HCV prevalence of 77.2% among this group [60].

#### *PWID* (1 study)

A study conducted on PWID attending an inner-city accident and emergency department showed high levels of morbidity and HCV exposure (74%) [61] (Table 2).

**Table 2:** Epidemiology post 2008

	Date and author	Setting	Sample (n)	Data collection	Design	Main results
Prisoners						
[13]	2014 Drummond et al	National Multi-site Prisons (15)	Prisoners (817; m = 772; Hx IDU = 26%)	2011	Observational Cross-sectional prevalence study including self- administered drug and risk questionnaire	Anti-HCV prevalence = 13% Significant risk factors (*p* < 0.05): • Hx of IDU and sharing drug taking equipment • Older age • female gender • prison tattoo
[57]	2014 Galander	Dublin Single site Prison	Male prisoners on MMT (119)	October 2011	Patient Survey Retrospective chart review	Anti-HCV = 38% • Anti-HCV negative = 33% • Unknown HCV status = 29% • Number anti-HCV on treatment = 0
PWUD						
[62]	2014 McCormick et al.	Dublin Single site Drug treatment clinic	Drug Users fibroscaned attending MMT (84; m = 66)		Letter – reporting on five year follow up study	Anti-HCV = 74.4% HCV-RNA = 58.3%, Heavy alcohol use = 37% Five-year mortality = 15% (Liver related deaths = 6; probable liver related = 1; drug overdose = 5; laryngeal carcinoma = 1) • 5/6 patients who died of liver disease, were HCV RNA-positive with heavy alcohol use • Mean liver stiffness values were higher in patients who died compared with survivors (28.5 kPa ±7.9 vs. 9.0 ± 1.5, *P* = 0.0045) • Mean liver stiffness values were higher in patients with liver-related death compared with survivors (50.6 ± 11.2, *P* < 0.0002). • 12 patients with fibroscan score > 14 kPa, (7 died; 4 developed liver failure) • Fibroscan scores were higher in patients with a history of heavy alcohol use (23 ± 4.5 vs. 5.6 ± 0.3, *P* < 0.0001). • A single liver stiffness measurement was highly predictive of liver-related mortality
[58]	Ryan and Ryan 2014	Limerick Single site Community drug treatment centre	Drug users on MMT(174)	2010–2012	Retrospective prevalence study (log review)	Anti-HCV 2010 = 6% Anti-HCV 2011 = 13% Anti-HCV 2012 = 24% On average 58 HCV tests ordered annually
	2014 Horan	Cork Single site Community drug treatment Centre	Drug users on MMT (30)	2-week periods ×2 separated by a few months in 2014	Chart audit of HCV screening measuring the effect of chart labelling (star)	• No evidence of HCV screen = 50% • Follow up audit = 72.7% had evidence of documentation of an HCV screen
	Burke M. Audit of HCV screening using retrospective patient records [Unpublished audit]	Dublin Multi-site Community drug treatment centres (23)	PWUD - Opioid users on MMT (358; 40% of eligible population; PWID = 79%)	2015	Audit of HCV screening using retrospective patient records	Anti-HCV = 66% HCV RNA = 65% • Screening uptake = 95% • RNA tested = 208
[63]	2016 McCombe et al.	Dublin Multi-site GP (16)	PWUD attending primary care for MMT (106)	2015	Commentary (letter) Secondary analysis of data collected during a feasibility study of an alcohol brief intervention for patients attending primary care for MMT	Anti-HCV = 51% • HCV tested = 99% • Self-reported HCV treatment = 19% Problem alcohol use = 45% • 37% were anti-HCV and had problem alcohol use Patients’ knowledge of HCV care can best be optimised through community-based approaches to HCV treatment
[59]	Keegan et al. 2017	Dublin Single site Community drug treatment centre	PWUD attending for MMT (228; m = 168)	January 2015	Cross sectional prevalence study (retrospective chart review) with associated risk factors	Anti-HCV = 63.6% with no significant gender difference (*p* = 0.717) Significant risk factors for HCV infection (*p* < 0.05): • age • age of first drug use • age of first injection • type of first drug used • early age of MMT entry Those with no IDU had decreased odds of being HCV positive by 91.1%.
	(Burke M: Audit of HCV screening using retrospective patient records, unpublished)	Dublin Multi-site Community drug treatment centre (23)	PWUD on MMT (282; PWID = 79%)	2016	Audit; Retrospective chart review	Anti-HCV = 79% HCV RNA = 65%
[60]	2017 Murtagh et al.	Dublin GPs Multi-site (*n* = 14)	Drug users on OST (133; m = 81)	2017	Cross-sectional Prevalence	Anti-HCV = 77.2% • 92.5% had been screened for HCV • 14 (14.7%) patients previously diagnosed with HCV had ever initiated HCV treatment
[9]	2017 Garvey et al	National	General population > 18 years (3795; m = 1860)	2014–2016	Anonymised and randomised laboratory analysis of residual serum samples at the NVRL	Anti-HCV = 1.4% HCV RNA = 0.57% (95% CI: 0.40–0.81%) • higher in men (0.91%; 95% CI: 0.61–1.4%) • east of the country (1.4%; 95%CI: 0.99–2.0%) • 0–39 years (1.1% (95% CI: 0.59–2.0%) • 40–49 years (1.1% (95% CI: 0.64–1.9%) • Men born between 1965 and 1984 from the east of the country have the highest rate of chronic HCV infection.
PWID						
[61]	2014 O’Connor et al	Dublin Single site Hospital ED	PWID (146; m = 101)	January – March 2010	Prospective observational study	Anti-HCV = 74% • High levels of comorbid illness
[19]	2016 Carew et al.	National	PWID registered on NDTRS (14,320; m = 10,597)	1991–2014	Mathematical modelling	Number of PWID with HCV infection = 12,423 (95% CI 10,799-13,161) Number of PWID with chronic HCV infection = 9317 (95% CI 8022-9996) • Estimated number of new infections peaked in 1997. • By 2014, more than one quarter (27.0%) of PWIDs with chronic HCV infection were estimated to have been infected for 0–10 years, 43.4% for 11–20 years, 22.8% for 21–30 years and 6.7% for over 30 years.

#### HCV risk factors

A total of 13 studies (1995–2017) reported on risk factors for HCV acquisition in both prisoner and PWUD populations. Prisoner risk factors identified were; a history of IDU, sharing needles while incarcerated, sharing drug taking equipment, older age, female gender, a previous history of incarceration and a history of having a prison tattoo. [13, 64, 65]. For drug users attending community-based OST risk factors identified were; a history of IDU, increased total number of lifetime injecting, closer social relationships with PWID, injecting in the homes of other PWID, a history of imprisonment, older age, male gender, drug use prior to 1989, co-infected with HIV or HBV, younger age of first drug use, first IDU and entry to MMT and type of drug first used [6, 17, 36, 37, 39, 42, 48, 51, 53, 59].

#### HCV screening uptake

A number of the epidemiological studies reported on HCV screening uptake among the study population. The first was a 2001 multi-site randomised chart review of 715 patients attending MMT. This study reports a screening uptake of 60%. The larger 2005 Dublin based multi-site study involving 1459 PWUD reported an 88% uptake of HCV screening.

A single site study from the NDTC reports very high levels of HCV screening uptake and an anti-HCV prevalence of 81.6% among study participants [40]. However only a third of anti-HCV patients were RNA tested showing a chronic HCV prevalence of 15.6%. This study also found high levels of accuracy for HCV self-report [40].

A 2003 study involving 531drug users attending MMT at 42 general practice locations reported that over two thirds had an HCV screen [42]. Two large HCV screening audits, identified through the grey literature search, reported high levels of HCV screening (Burke M: Audit of HCV screening using retrospective patient records, unpublished),(Burke M. Audit of HCV screening using retrospective patient records [Unpublished audit]). Dublin. Uptake of HCV screening was less than in Dublin clinics (50%) (Horan, 2014; Ryan & Ryan, 2014) The Cork (second largest city in Ireland) study identified that chart labelling (starring) had a positive impact on the uptake of screening among this patient cohort (Horan A: Chart audit of HCV screening measuring the effect of chart labelling, unpublished). The most recent prevalence study in PWUD attending OST in general practice reported high uptake of HCV screening (92.5%) [60]. A later 2007 multi-site study reported high levels of HCV screening among PWUD on MMT in Dublin based general practice (77%) [44]. Only a third of those infected had been screened for chronic infection (36%) [44].

#### Identified gaps

No studies reported on incidence infections specifically in prisoners, PWUD or PWID in general practice or not on OST and PWUD without a history of IDU. Only two studies reported on HCV prevalence in PWUD outside of Dublin and both where from secondary urban centres. The majority of the prevalence studies are over a decade old and only report on anti-HCV prevalence and not on HCV-RNA prevalence which limits their usefulness at estimating the levels of chronic untreated infection and re-infection. The most recent epidemiological studies included in this review are mostly chart review audits which limit their usefulness to inform policy and strategy.

#### Guidelines and policy (8 studies)

A total of 8 documents were identified that fitted into this category. The first published national document relating to HCV infection in PWUD and prisoners was published in 2004 [66]. This was a regional (Dublin-based) document that reported on the views of 70 stakeholders (service planners, funders, providers and users) on a broad range of HCV related issues including policy and clinical issues [66].

In the same year, a research lead initiative developed consensus guidelines on HCV management in general practice [67]. The effectiveness of these guidelines was later evaluated in a cluster randomised trial and were found to improve the uptake of screening in practices where the policy was implemented (OR = 3.76 CI = 1.3–11.3) [56].

The National Hepatitis C Strategy 2011–2014 was the first published strategy relating to all those infected with HCV in Ireland [14]. The strategy spans surveillance, prevention, screening and treatment of HCV infection and reports on both groups (PWUD and prisoners). The Department of Health (DOH) subsequently published a report: A Public Health Plan for the Therapeutic Treatment of Hepatitis C in 2015 which recommended the establishment of the National Hepatitis C Treatment Programme (NHCTP) in the HSE [68].

The NHCTP was established in 2014 and oversees treatment provision to all HCV infected patients in Ireland, including PWUD and prisoners [26]. This program has a number of specialist committees that advise on the treatment criteria and guidelines and also includes a treatment register which collects data on treatment uptake and outcomes.

The HSE developed national HCV screening guidelines to identify HCV-infected individuals who are currently unaware of their HCV status [10]. These guidelines were preceded by two opioid substitution guidelines that contained recommendations on the screening of drug users on OST [69].

The Irish Prison Services are responsible for the provision of health care in all prisons in the ROI and in parallel to community-based guidelines have further sets of guidelines that deal specifically with HCV management within the IPS [70]. The provision of all healthcare, including HCV treatment is guided by the international principle of community equivalence of care [71].

The major gap identified in this category is that the national strategy was published pre-DAA and no longer reflects the reality of HCV treatment in particular the challenges related to moving HCV treatment from hospital services to the community. The new national HCV screening guidelines provides a clear road map on who to screen but to date there is not data on the success of their implementation. It is also important that the IPS update their health care standards in line with the new national screening guidelines and NHCTP to ensure community equivalence (Table 3).

**Table 3:** Guidelines and policy

	Date and author	Setting	Sample (n)	Data collection	Design	Main results
[67]	2004 Dublin Area Hepatitis C Initiative Group	ERHA	GPs	2001–2002	Descriptive study reporting on HCV management guideline development for GPs	The guidelines cover advice to GPs on all aspects of care of patients at risk of HCV, including • general and preventative care • care of other bloodborne and hepatotoxic viruses • factors to be considered and appropriate evaluation prior to referring a patient for assessment at a hepatology unit.
[66]	2004 Hepatitis C Scientific Advisory Subgroup of the Blood Borne Virus Forum and the Eastern Regional Health Authority	ERHA	16 workshops with service planners, health and social care professionals and service users (70	2004	A regional hepatitis C strategy document using open space technology	Reported on: • health promotion • role of the media • service provision • research, policy and planning • education and training, • liaison • key workers • co-ordination and collaboration • accessing services • psychological and complementary therapies
[72]	2006 Long	National	Drug users	1995–2005	Review	Reports on • 9 prevalence studies • 7 studies identifying risk factors
[70]	2009 IPS	Dublin	Prisoners	–	Guidelines	Provide prisoners • general health information • advice and testing • referral to appropriate specialist services in relation to HCV where clinically indicated • treatment and support for those chronically infected.
[14]	2012 HSE	National	PWID & prisoners	2007	First national strategy	• Reviewed and updated recommendation from the 2004 ERHA report. • Developed 36-point action plan Prioritising recommendations for 2011–2012 in the areas of HCV surveillance, education, prevention and treatment.
[68]	2014 DOH	National	HCV infected patients	–	Guidelines	Recommendations: • HSE establish a Hepatitis C Treatment Programme with a strong governance and management structure • Provide drug treatment to those with greatest clinical need as a priority and treat as many patients as possible with the available resources
[69]	HSE 2017	National			OST Guidelines	• All drug users (including non-PWID) should be screened for HCV. • Anti-HCV patients should be tested for HCV-antigen and LFTs • all antigen positive patients should be referred to specialist services for PCR, fibroscanning and consideration for treatment. • All patients at risk of HCV infection should be given information and advice on the disease and how it is transmitted. • IDU and alcohol misuse should not exclude patients from treatment. • Risks of concurrent alcohol use should be explained to anti-HCV patients.

#### Treatment outcomes (13 studies)

A number of papers reported on treatment uptake and outcomes. These studies found poor engagement with hepatology services for liver assessment and treatment by PWUD [43, 44, 55, 60, 63, 73]. Predictors for poor treatment follow up were, younger age, active IDU and advanced HIV disease, male gender [74, 75]. A single prison study reported that no HCV infected prisoner on MMT was on HCV treatment at the time of the study [57].

A number of studies reported SVR from interferon -based treatment and found SVR ranging from (53–74%) with PWID having similar outcomes to other patients [74–77].

In-reach hepatology services provided HCV treatment at two Dublin based prison location. An audit of these sites shows high compliance with treatment and good treatment outcomes [78].

A single study reported on the use of tele-medicine as a novel approach to increasing treatment among HCV infected PWUD attending MMT. This study showed high levels of satisfaction among professionals engaging with the initiative [79].

A number of gaps in the treatment outcome literature were identified including: the lack of SVR outcomes for DAA treatment, re-infection rates and treatment uptake and completion rates. There are no mathematical modelling studies conducted using existing surveillance data and treatment uptake and outcomes to map pathways towards HCV elimination in Ireland (Table 4).

**Table 4:** Treatment outcomes (7 studies)

	Date and author	Setting	Sample (n)	Data collection	Design	Main results
[50]	2005 Keating et al	Dublin Multi-site 5 drug treatment centres	PWID mono-infected with HCV (496; m = 341; 2 years follow up = 127)	Jan 1997- Nov 2001 Repeat follow up testing in 2003	Cross-sectional prospective study on HCV clearance	HCV RNA: • Negative = 38% (self-clearance) (f = 47.4%; m = 34.5%) • Follow up (2 yrs) = 82.2% sustained viral clearance • Overall viral clearance = 31.1% • Genotype distribution (1 = 48.8%; 3 = 48.5%)
[76]	2006 Hopkins	Single site Hospital	Co-infected with HIV and HCV patients with CD4 counts > 200 cells/mL (45; m = 39; PWID = 58%)	June 2001–2003	Open-label, prospective study	SVR = 53% • Genotype 2 and 3 patients had a significantly higher SVR (75%) than genotype 1 (19%) • Adverse events occurred frequently
[74]	2011 Kieran et al	Dublin Single-site Hospital Integrated HIV/HCV clinic	Co-infected attendees (386; m = 278)	October 2008–January 2009	Retrospective chart review	202/386 – referred to co-infected clinic, with 107 completing treatment • SVR = 44% (similar outcomes for PWID compared to patients with other transmission risks) Associations with missed appointments • younger age • active IDU • advanced HIV infection Dedicated co-infection clinics lower the threshold for treatment and improve management of liver disease in co-infected patients
[75]	2011 Lowry et al	Dublin Single-site Hospital	HCV mono-infected patients referred (588 individuals (repeat referrals = 742 cases); m = 388; PWID = 74%)	2000–2007	Retrospective chart review	SVR = 74% • History of IDU was not a significant predictor of lower therapy completion rate or achievement of SVR In total, 451 (61%) dropouts occurred • 141 (19%) failed to attend their initial appointment • 180 dropped out from early outpatient management • 29 failed to attend liver biopsy • 81 defected from subsequent outpatient follow-up. Statistically significant associations with history of injection drug use • dropout immediately after the referral (*P* < 0.001) • dropout from early outpatient management (P < 0.001) • dropout over entire span of disease management (P < 0.001) Male sex was also associated with dropout from disease management (*P* < 0.05)
[52]	2012 Kelly and Kelly	Dublin Single site GP	PWID (82; m = 62%)	1985–2010	Longitudinal cohort study	Anti-HCV = 33% at 10 yrs. (survivors) Anti-HCV = 40% at 25 yrs. (survivors) • 63% of the cohort had died by 2010, of which 26 were attributed to HIV disease • Median survival time for those ant-HCV = 21 years (95% CI 15.5–26.5) which was significantly lower than the median survival time for drug users with a negative hepatitis C status. (*p* = 0.006)
[77]	2017 Elsherif et al	Dublin Single-site Hospital	HCV infected patients (1000; Former PWID (> 6 months) = 608; Recent PWID (< 6 months) = 85; Non-drug users = 307)	2002–2012	Retrospective chart review	SVR in PWID = 64.2% • No significant compared to non-PWID (60.9%) [RR = 1.05, 95% CI 0.95 ± 1.17] • There was no significant difference in SVR rates between the groups controlling for genotype (48.4% vs 48.4% for genotype 1; 74.9 vs 73.3% for genotype 3). • No significant difference in treatment non-adherence between the groups (8.4% in PWID vs 6.8% in non-PWIDs; RR = 1.23, CI 0.76 ± 1.99) • Former and recent PWID had similar adherence rates.

#### HCV -related health issues (8 studies)

A number of studies reported on HCV related health issues. Two hospital-based studies found higher levels of depression and anxiety among HCV infected patients whose risk factor for acquisition was IDU [80, 81].

A 2003 mathematical modelling study estimating the national HCV- related disease burden over 20 years, predicted 1214 cases of cirrhosis, 35 cases of HCC, 60 cases of hepatic decompensation and 50 cases of liver related deaths per annum [49].

A number of papers describe high levels of alcohol use among HCV infected PWUD (41%) [40, 62, 63]. Studies report high levels of HCV-related liver disease and awareness that excessive alcohol had a negative impact on HCV disease progression among PWUD on OST [62, 73] [40].

A 25 year follow up study of PWID attending general practice found high levels of mortality in a cohort with HCV exposure having a negative impact on life expectancy (O’Kelly & O’Kelly, 2012). A unique five-year follow up study among PWUD attending OST showed high mortality rates of among patients with high Fibroscan scores which was associated with heavy alcohol use. The study concluded that a single Fibroscan score was highly predictive of mortality (McCormick et al., 2014).

Overall the research on HCV related health issues was considered to be scant and incomplete. While a number of studies report on levels of liver disease in small groups of PWUD on OST, the literature does not provide a clear picture of the HCV disease burden in these two groups. In the early days of DAA treatment HCV infected patients with advanced liver disease were prioritised for treatment access but it is unclear how many of these accessed or completed treatment. There is also no published data on the numbers of HCV infected PWUD who die annually from non-HCV related causes. This may be considerable given the high levels of drug related deaths in Ireland and if not accurately quantified will have an impact on Irish HCV elimination strategies (Table 5).

**Table 5:** HCV-related health issues

	Date and author	Setting	Sample (n)	Data collection	Design	Main results
[80]	2001 Goulding et al	Dublin Single-site Hospital	Patients with chronic HCV infection (77; m = 17; PWID = 25)		Cross-sectional Prevalence of rheumatological disease, anxiety and depression and relationship to mode of acquisition	Anxiety and depression scores were significantly higher in IVDUs (*P* = 0.005) compared with controls.
[82]	2005 Golden et al	Dublin Single-site Hospital	HCV infected patients awaiting interferon treatment (90; m = 67; 47% PWID)		Prevalence of mood disorder and associated risks using a self-completed structured questionnaire	Depressive disorders: • 1-month prevalence = 21% (72% previously undiagnosed) • Current MMT strongly associated with risk of depression (OR, 5.0; 95% CI, 1.08–23.0). • After adjustment for age and sex, depression was associated with poorer work and social adjustment, lower acceptance of illness, higher illness stigma, poorer reported thinking and concentration, and higher levels of subjective physical symptoms (all *P* < .05) Anxiety disorders: • 1-month prevalence = 24% (86% previously undiagnosed) • Anxiety disorders were uncorrelated with any risk factor.
[78]	2018 Mc Kiernan et al	Prisons Multi-site Community Single site (hospital)	HCV infected prisoners referred for HCV treatment (510; Treatment outcomes = 104)	2010–2018	Retrospective record audit Comparisons between community and prison populations	SVR prison = 90.3% SVR community = 87.5% Referrals: • Mountjoy = 265 • Wheatfield = 173 • Midlands = 33 • Portlaois =15 • Limerick =6 • Others =11

#### Qualitative research reporting on patients’ and health providers’ experiences

A number of studies report a high awareness of HCV infection among PWUD on OST both in primary care and drug treatment centres [40, 43]. This includes the knowledge that IDU is a major risk factor for transmission among this group and the health implications of being infected [43]. There were also high levels of awareness of patients own HCV status, of HCV harm reduction measures, and adverse effects alcohol has on the progression of HCV liver disease [40, 43].

A prison–based study exploring the health needs of female prisoners using focus group methodology reported that HCV infection was identified as a health concern among Irish female prisoners [25].

A single study reported on negative patient experiences with regard to testing, assessment and treatment [43]. HCV related stigma was reported in many of the qualitative studies [25, 43, 73, 81–84].

A number of studies identified barriers to PWUD engagement in HCV treatment including; ongoing alcohol and drug use, fear of HCV treatment and liver biopsy, imprisonment, distance to hospital, early morning appointments, perceptions of HCV infection as relatively benign, fear of investigations and treatment including liver biopsy and interferon, feeling well, limited knowledge of testing sites, not being referred for specialist investigations, ineligibility for treatment and competing priorities (employment, education, and addiction) [43, 73, 79, 83]. Some of these studies reported on enablers to PWUD engagement which included; afternoon appointments, enhanced prison referral mechanisms into the community, community fibroscanning, location of services within the addiction treatment services, relationships with health care providers, trust in providers, concern for the service-user, continuity of care, education on HCV infection, investigations, and treatment, becoming symptomatic, responsibilities for children, wanting to move on from drug use [73, 83].

Only two studies reported on healthcare professionals’ experience of providing care to HCV infected prisoners and PWUD. A single prison study reported lack of knowledge regarding BBVs including HCV had a negative impact on prison officers’ work [85]. A recent study identified lack of time and inadequate funding as barriers to community GPs engaging with HCV treatment [79].

As previously identified the major deficit in this category is that it reports from a pre-DAA era. This is particularly relevant for barriers to treatment engagement which was related to the fear and experience of side effects of interferon- based treatments. There is a need to conduct more up to date quantitative research on PWUD S′ and prisoners’ experience of the HCV treatment cascade with DAA treatments. This can best inform the planning of effective HCV delivery systems (Table 6).

**Table 6:** Qualitative research reporting on patients’ and health providers’ experiences

	Date and author	Setting	Sample (n)	Data collection	Design	Main results
[43]	2005 Cullen et al	Dublin Single site General practice	Heroin users - past history (25)	2002 (6 months)	Mixed methods using interviewer-administered semi-structured questionnaire	Anti-HCV =88% • Follow up investigations for HCV =8 • Treatment = 1 • 100% aware of HCV • 22/25 consulted healthcare professional about HCV • 21/25 knew HCV infection was caused by injecting • High awareness of harm reduction measures and health implications of HCV • Negative experiences of diagnosis, assessment and treatment
[85]	2005 Dillon	Dublin Multi-site 4 prisons	Prison officers		Cross sectional survey	• 87% reported not knowing enough about these diseases to enable them to take the necessary precautions at work • Longer serving and senior officers were less fearful and less anxious about contracting the infections • Officers who had received hepatitis B vaccination were no less worried about hepatitis B than unvaccinated colleagues • Training on blood borne viruses had little effect on prison officers’ knowledge or perception of blood borne viral infections
[81]	2006 Golden	Dublin Single-site Hospital	HCV infected patients awaiting treatment (87; m = 64; PWID = 46%)		Prevalence of illness-related stigma and mood disorders using standardised instruments	• Fear of disclosure combined with social isolation and social rejection • Stigma was higher in those in manual occupations and the unemployed than in those in non-manual occupation • High levels of disease-associated stigma in those with disease associated with IDU and iatrogenic disease caused by transfusion or anti-D blood products • Stigma was associated with depression (OR = 1.4) • Stigma was also associated with poorer work and social adjustment, lower acceptance of illness, higher subjective levels of symptoms and greater subjective impairment of memory and concentration. These associations were replicated in the non-depressed subsample. • Strong link between stigma and well-being in hepatitis C
[83]	2010 Swan et al	Dublin Multi-site (7) Drug treatment clinic (2) GP (1) Community drop in (1) Hepatology (2) ID clinic (1)	PWID (36; m = 28)	2007–2008	In-depth one-to-one interviews using grounded theory methodology	Anti-HCV = 91% HIV co-infected = 11% Barriers to HCV screening and treatment: • Perceptions of HCV infection as relatively benign • fear of investigations and treatment including liver biopsy and interferon • feeling well • limited knowledge of testing sites • not being referred for specialist investigations • ineligibility for treatment • competing priorities (employment, education, and addiction). Facilitators to HCV screening and treatment: • relationships with health care providers • **t**rust in providers • concern for the service-user • continuity of care • education on HCV infection, investigations, and treatment • becoming symptomatic • responsibilities for children • wanting to move on from drug use
[84]	Whitaker et al. 2011	Dublin	Drug using sex workers (35; m = 4)		One-to-one in-depth interviews	Multiple layers of stigma were reported, linked to sex work, drug use (including IDU) and having contracted HIV or HCV • Stigma was powerfully reinforced by the language routinely used by health professionals.
						• To improve the effectiveness of harm-reduction interventions, it is recommended that service providers change their language, in particular in recognition of the human dignity of these clients, but also to help attract and retain drug users in services, and to help reduce the unacceptable mortality levels among drug users.
[73]	2017Crowley et al	Dublin Single site Community drug treatment centre	PWID attending community fibroscan clinic (68)	2017	Mixed methods Researcher administered questionnaire	Attendance = 90% • high levels of unemployment (90%) and homelessness (40%)
						• higher fibroscan scores (> 8.5Kpa) were associated with longer time since diagnosis (*p* = 0.016).
						Patient identified barriers to engagement:
						• alcohol and drug use
						• fear of HCV treatment and liver biopsy
						• imprisonment
						• distance to hospital
						• early morning appointments.
						Patient identified enablers: • afternoon appointments
						• enhanced prison referral mechanisms into the community Fibroscan unit
						• location of services within the addiction treatment and detoxification services
[79]	2017 Ni Cheallaigh et al.	National Multi-site Community drug treatment centres, homeless hostels, GPs	Study sites: Pilot sites (4): 2 Dublin based community drug treatment centres, 1 Waterford based; 1 Dublin based homeless hostel 10 interviewees from 8 sites at baseline. 6 participants in pilot programme at study completion	Mar-Oct 2015	Purposive sampling	Estimated HCV prevalence in GP practices = 1–10%
						Estimated chronic prevalence in pilot sites = 15–75%
						• PWID were identified as the main group facing barriers to accessing specialist HCV care.
						• State-employed doctors and nurses were successfully recruited to participate in the project.
						• GPs did not participate, due mainly to a lack of time and the absence of reimbursement for participation.
						• Benefits to practitioners and their patients were reported. Participants expressed interest in continued engagement with similar multidisciplinary, multisite educational interventions in the future.

## Discussion

Overall this review found a larger than expected quantity and greater scope of literature published on HCV infection in Irish prisoners and PWUD. The literature was both quantitative and qualitative, employed many different methodologies and covered a 35-year period from 1983 to 2018.

The majority of the studies are epidemiological and report on HCV prevalence in both cohorts. This scoping review found that Ireland has low rates of HCV infection in the general population compared to other European countries [5, 27, 86, 87]. The most up to data (residual sera study) showed a reduced prevalence compared to a 2012 mathematical modelling study [6, 9]. The different rates could be explained by the differing methodologies or by a reduction in new infections. The absence of a population prevalence study makes it difficult to have a true HCV population estimate and impacts on the development of strategies to identify the undiagnosed population [6, 88].

This review found that IDU is the most common risk factor for HCV acquisition in Ireland and that the prevalence of HCV infection in Irish prisoners and PWUD is much higher than the general population. The prison-based studies included in the review found a reducing HCV prevalence with the most recent study (2014) reporting a prevalence of 13% [13]. This is much lower than the global estimates of 25% [11] but similar to other European country estimates [5]. The prevalence of HCV in all groups of PWUD and PWID is over 50%. Again, this is in keeping with other global and European estimates for this group [2, 5]. The majority of the studies included in the review are over a decade old, only include drug users on OST and all but two are Dublin based. There is a paucity of up to date literature on HCV prevalence in PWUD from outside Dublin and none at all on PWUD not on OST. A small number of studies reported on rates of chronic infection, but no study reported on the population based chronic HCV prevalence. This is a significant deficit in the literature as it does not allow for accurate estimates of untreated HCV infections and re-infections [89].

This review also found low levels of RNA testing for those identified as anti-HCV positive. Consideration should be given too adapting a reflex-RNA testing approach to HCV screening in Ireland [10]. This has the potential to reduce the number of bloods required in a group where venepuncture can be difficult and an indemnified barrier to engagement in HCV care [90]. As Ireland scales up HCV screening and treatment it is important that accurate data on levels of active HCV infection is available to optimise and evaluate the effectiveness of the national HCV strategy.

This review identified significant strategy, policy and guideline documents published over the last 14 years related to HCV. These provide a broader national context in which to interpret the literature and allow for comparisons to made with international and European strategies aimed at HCV elimination [27, 28]. However, it is concerning that the national strategy was published in 2012 and much of its content relates to interferon-based treatments and related challenges [14]. While the NHCTP provides up to date recommendations on HCV treatment [26], a new strategy is required to support the upscaling of HCV care, in particular, its movement into community addiction services and primary care. The national screening guidelines provide a useful guide to active HCV case identification [10]. These guidelines need to be underpinned with adequate resources and an evaluation to measure their effectiveness in identifies what is considered to be a significant level of hidden HCV infection in Ireland [6].

A handful of studies in this review report on treatment uptake and outcomes and on the lived experience of Irish HCV infected prisoners and PWUD [43, 44, 57, 83]. The evidence suggests poor uptake by HCV infected community PWUD with hospital-based services. This review also identified the many barriers related to HCV treatment uptake by this group [73, 83]. Many of these barriers are associated with previous interferon-based treatment and will be eliminated by the availability of DAA [29, 30]. This review also reports a number of facilitators to HCV engagement by Irish PWUD including the movement of HCV treatment out of specialised hospital services into community OST clinics. The success of this approach has been reported previously in the literature [91, 92] and is now the strategy being adapted the NHCTP [26]. This review found that treatment outcomes were not negatively impacted by patients having a history of IDU, however active drug use was identified as having a negative impact on treatment engagement. These findings need to be viewed with caution since they are related to interferon-based treatments [77]. Many patients were excluded from accessing treatment based on; a history of active IDU, excess alcohol or other drug use, concerns regarding relapse or mental health issues [91, 93]. Treatment was also only provided in specialist hospital services and only imitated after many hospital visits. This ensured that only the most stable and compliant PWUD accessed treatment. Broadening treatment eligibility. Increasing treatment locations and the use of short acting pangenotypic DAA will engage many less stable PWUD with HCV care [29, 30]. It is important the real-world data is available to monitor SVR post treatment and re-infection rates [94]. Similar to other published studies this review found SVR rates in prisoners equal or better that in the community [95, 96]. But challenges remain in screening and engaging more prisoners in HCV care [97]. This review found no studies reporting on prison-based barriers and facilitators to HCV care.

There is very little data available on the levels of HCV related liver disease in PWID and prisoners [98]. Where available, the evidence supports increasing levels of morbidity and mortality in these groups [98, 99]. This review found that HCV infection was associated with increased mortality and that problem alcohol use was common among HCV infected PWUD. Irish epidemiological studies report that there is an aging cohort of HCV infected PWUD, the majority having the infection for over 10 years. It is reasonable to expect that there is significant related liver disease in this cohort. While this group where initially prioritised for DDA treatment there is no data on how many of this high-risk group remain untreated. Studies on rates of HCV related liver disease and treatment uptake in this high-risk groups are required to ensure that are treatment strategies are successful in reducing HCV related morbidity and mortality.

Noticeable gaps identified in the literature on HCV in Irish prisoners and PWUD was the lack of studies, on strategies to prevent HCV infection and rates of incident infection in both groups. Ireland has a well-established network of harm reduction services including OST and needle exchange programs which are known to be effective in reducing HCV infection if provided with adequate coverage [100, 101]. However, there is no data available on their effectiveness in an Irish setting. Monitoring the rates of new infections will provide an indicator to the success of harm reduction services and the impact of treatment as prevention as more people are successfully treated with SVR.

## Conclusion

This review summarises the literature on HCV in Irish PWUD and prisoners. The findings show that in Ireland, 0.5% of the general population have chronic HCV infection (60% undiagnosed), 13% of prisoners and over 50% of PWUD on OST have been exposed to HCV infection. People with a history of injecting heroin carry the greatest HCV disease burden in Ireland, with the majority having the infection for greater than 10 years. Increasing HCV related morbidity and mortality in this cohort, particularly in those with problem alcohol use is a concern. Irish PWUD engage poorly with hospital-based services and many barriers to HCV treatment uptake in this cohort have been identified. HCV treatment outcomes in Irish PWID is similar to other patient groups. In-reach hepatology services to Irish prisons has a positive impact on both uptake and successful completion of HCV treatment and its expansion to all prisons in Ireland should be considered. This review identified a range of policy and strategy documents that can inform national HCV screening, treatment and prevention programmes. A number of gaps in the literature were identified including; lack of reliable national prevalence data on untreated HCV infection (general population, PWUD and prisoners), accurate estimates of HCV related disease burden, HCV incidence infection in both PWUD and prisoners, barriers and facilitators to prisoners engaging with HCV services and HCV treatment uptake and outcomes in both groups. The lack of HCV epidemiolocal data on PWUD outside of Dublin and those not on OST is a concern. It is also important that policy and strategy documents are kept current to reflect international evidence-based practice and are informed by more accurate HCV surveillance data. This review is timely as Ireland scales up community-based HCV screening and treatment and gathers the available literature on HCV in Irish PWUD and prisoners. It is a useful starting point and can be used as a baseline to measure our efforts to eliminate HCV infection as a major Irish public health problem.

## Additional file

Additional file 1: Search strategy and excluded studies. This file shows an example of the search strategy used for PubMed and the bases on which studies were excluded from the Scoping Review. (DOCX 25 kb)

## Data Availability

Not applicable.

## Abbreviations

BBV: Blood-borne virus
CI: Confidence interval
CTL: Central Treatment List
DAA: Direct-acting antivirals
DOH: Department of Health
DOJ: Department of Justice
EMCDDA: European Monitoring Centre for Drugs and Drug Addiction
ESLD: End stage liver disease
GPs: General practitioners
HCC: Hepatocellular carcinoma
HCV: Hepatitis C Virus
HSE: Health Service Executive
HSPC: Health Surveillance Protection Centre
IDU: Injecting drug use
IPRT: Irish Penal Reform Trust
IPS: Irish Prison Service
MMT: Methadone maintenance treatment
NDTC: National Drug Treatment Centre
NHCTP: National Hepatitis C Treatment Programme
NVRL: National Virus Reference Laboratory
OR: Odds ratio
OST: Opioid substitution treatment
PWID: People who inject drugs
PWUD: People who use drugs
ROI: Republic of Ireland
SVR: Sustained virologic response
