# Supplementary material for: Hepatitis C virus infection in Irish drug users and prisoners – a scoping review
Source: BMC Infect Dis. 2019 Aug 8;19:702. doi: 10.1186/s12879-019-4218-6 (PMC6686252; doi:10.1186/s12879-019-4218-6)
Supplement: Supplementary file 1 — Search strategy and excluded studies. This file shows an example of the search strategy used for PubMed and the bases on which studies were excluded from the Scoping Review. (DOCX 25 kb) [file 12879_2019_4218_MOESM1_ESM.docx]

Appendix 1 : Sample search (Pubmed) searched on 20/07/2018 and example of study selection based on inclusion and exclusion criteria

#1: (("hepatitis c"[MeSH Terms] OR "hepatitis c"[All Fields] OR "hepacivirus"[MeSH Terms] OR "hepacivirus"[All Fields]) AND (("prisoners"[MeSH Terms] OR "prisoners"[All Fields]) OR ("prisons"[MeSH Terms] OR "prisons"[All Fields]) OR ("drug users"[MeSH Terms] OR ("drug"[All Fields] AND "users"[All Fields]) OR "drug users"[All Fields]))) AND ("ireland"[MeSH Terms] OR "ireland"[All Fields])

Results: 57

| **Study** | **Included (Yes/No)** | **Reason for exclusion** |
| --- | --- | --- |
| Swan D, Cullen W, Macias J, Oprea C, Story A, Surey J, Vickerman P, Lambert  JS. Hepcare Europe - bridging the gap in the treatment of hepatitis C: study  protocol. Expert Rev Gastroenterol Hepatol. 2018 Mar;12(3):303-314. doi:  10.1080/17474124.2018.1424541. Epub 2018 Jan 16. PubMed PMID: 29300496. | No | Study protocol |
| Williams R, Alexander G, Armstrong I, Baker A, Bhala N, Camps-Walsh G, Cramp  ME, de Lusignan S, Day N, Dhawan A, Dillon J, Drummond C, Dyson J, Foster G,  Gilmore I, Hudson M, Kelly D, Langford A, McDougall N, Meier P, Moriarty K,  Newsome P, O'Grady J, Pryke R, Rolfe L, Rice P, Rutter H, Sheron N, Taylor A,  Thompson J, Thorburn D, Verne J, Wass J, Yeoman A. Disease burden and costs from  excess alcohol consumption, obesity, and viral hepatitis: fourth report of the  Lancet Standing Commission on Liver Disease in the UK. Lancet. 2018 Mar  17;391(10125):1097-1107. doi: 10.1016/S0140-6736(17)32866-0. Epub 2017 Nov 29.  Erratum in: Lancet. 2017 Dec 7;:. PubMed PMID: 29198562. | No | UK based |
| Marshall AD, Cunningham EB, Nielsen S, Aghemo A, Alho H, Backmund M, Bruggmann  P, Dalgard O, Seguin-Devaux C, Flisiak R, Foster GR, Gheorghe L, Goldberg D,  Goulis I, Hickman M, Hoffmann P, Jancorienė L, Jarcuska P, Kåberg M, Kostrikis  LG, Makara M, Maimets M, Marinho RT, Matičič M, Norris S, Ólafsson S, Øvrehus A,  Pawlotsky JM, Pocock J, Robaeys G, Roncero C, Simonova M, Sperl J, Tait M,  Tolmane I, Tomaselli S, van der Valk M, Vince A, Dore GJ, Lazarus JV, Grebely J;  International Network on Hepatitis in Substance Users (INHSU). Restrictions for  reimbursement of interferon-free direct-acting antiviral drugs for HCV infection  in Europe. Lancet Gastroenterol Hepatol. 2018 Feb;3(2):125-133. doi:  10.1016/S2468-1253(17)30284-4. Epub 2017 Oct 3. PubMed PMID: 28986139. | No | Not specific to study population |
| Crowley D, Cullen W, Laird E, Lambert JS, Mc Hugh T, Murphy C, Van Hout MC.  Exploring Patient Characteristics and Barriers to Hepatitis C Treatment in  Patients on Opioid Substitution Treatment Attending a Community Based  Fibro-scanning Clinic. J Transl Int Med. 2017 Jun 30;5(2):112-119. doi:  10.1515/jtim-2017-0017. eCollection 2017 Jun. PubMed PMID: 28721344; PubMed  Central PMCID: PMC5506411. | Yes |  |
| Elsherif O, Bannan C, Keating S, McKiernan S, Bergin C, Norris S. Outcomes  from a large 10 year hepatitis C treatment programme in people who inject drugs:  No effect of recent or former injecting drug use on treatment adherence or  therapeutic response. PLoS One. 2017 Jun 21;12(6):e0178398. doi:  10.1371/journal.pone.0178398. eCollection 2017. PubMed PMID: 28636638; PubMed  Central PMCID: PMC5479520. | Yes |  |
| Wiessing L, Ferri M, Běláčková V, Carrieri P, Friedman SR, Folch C, Dolan K,  Galvin B, Vickerman P, Lazarus JV, Mravčík V, Kretzschmar M, Sypsa V,  Sarasa-Renedo A, Uusküla A, Paraskevis D, Mendão L, Rossi D, van Gelder N,  Mitcheson L, Paoli L, Gomez CD, Milhet M, Dascalu N, Knight J, Hay G, Kalamara E,  Simon R; EUBEST working group, Comiskey C, Rossi C, Griffiths P. Monitoring  quality and coverage of harm reduction services for people who use drugs: a  consensus study. Harm Reduct J. 2017 Apr 22;14(1):19. doi:  10.1186/s12954-017-0141-6. PubMed PMID: 28431584; PubMed Central PMCID:  PMC5401609. | No | Not specific to study population or country |
| Swann RE, Mandalou P, Robinson MW, Ow MM, Foung SK, McLauchlan J, Patel AH,  Cramp ME. Anti-envelope antibody responses in individuals at high risk of  hepatitis C virus who resist infection. J Viral Hepat. 2016 Nov;23(11):873-880.  doi: 10.1111/jvh.12568. Epub 2016 Jul 13. PubMed PMID: 27405885; PubMed Central  PMCID: PMC5244678. | No | Not specific to study population or country |
| Sander G, Scandurra A, Kamenska A, MacNamara C, Kalpaki C, Bessa CF, Laso GN,  Parisi G, Varley L, Wolny M, Moudatsou M, Pontes NH, Mannix-McNamara P, Libianchi  S, Antypas T. Overview of harm reduction in prisons in seven European countries.  Harm Reduct J. 2016 Oct 7;13(1):28. Review. PubMed PMID: 27717368; PubMed Central  PMCID: PMC5055713. | No | Reports on data from a study already included in the scoping review (Drummond et al., 2014). |
| Hope VD, Cullen KJ, Smith J, Jessop L, Parry J, Ncube F. Is the recent  emergence of mephedrone injecting in the United Kingdom associated with elevated  risk behaviours and blood borne virus infection? Euro Surveill. 2016 May  12;21(19). doi: 10.2807/1560-7917.ES.2016.21.19.30225. PubMed PMID: 27195614. | No | UK based |
| Surah S, Adams R, Townsend L, Reynolds I, Kinahan JC, Keating S, Mulcahy F,  Keenan E, Barry M, Lyons F. Health-related quality of life of HIV-infected  intravenous drug users. Int J STD AIDS. 2013 Nov;24(11):867-74. doi:  10.1177/0956462413486454. Epub 2013 Jul 23. PubMed PMID: 23970601. | No | Reports on HIV infected drug users |
| O'Connor G, McGinty T, Yeung SJ, O'Shea D, Macken A, Brazil E, Mallon P.  Cross-sectional study of the characteristics, healthcare usage, morbidity and  mortality of injecting drug users attending an inner city emergency department.  Emerg Med J. 2014 Aug;31(8):625-9. doi: 10.1136/emermed-2012-201934. Epub 2013  Apr 27. PubMed PMID: 23625509. | Yes |  |
| Klimas J, Field CA, Cullen W, O'Gorman CS, Glynn LG, Keenan E, Saunders J,  Bury G, Dunne C. Psychosocial interventions to reduce alcohol consumption in  concurrent problem alcohol and illicit drug users: Cochrane Review. Syst Rev.  2013 Jan 12;2:3. doi: 10.1186/2046-4053-2-3. Review. PubMed PMID: 23311684;  PubMed Central PMCID: PMC3564788. | No | Does not report on study population |
| Klimas J, Field CA, Cullen W, O'Gorman CS, Glynn LG, Keenan E, Saunders J,  Bury G, Dunne C. Psychosocial interventions to reduce alcohol consumption in  concurrent problem alcohol and illicit drug users. Cochrane Database Syst Rev.  2012 Nov 14;11:CD009269. doi: 10.1002/14651858.CD009269.pub2. Review. Update in:  Cochrane Database Syst Rev. 2014;12:CD009269. PubMed PMID: 23152270. | No | Does not report on study population |
| Dunford L, Carr MJ, Dean J, Waters A, Nguyen LT, Ta Thi TH, Thi LA, Do HD,  Thi TT, Nguyen HT, Diem Do TT, Luu QP, Connell J, Coughlan S, Nguyen HT, Hall WW,  Nguyen Thi LA. Hepatitis C virus in Vietnam: high prevalence of infection in  dialysis and multi-transfused patients involving diverse and novel virus  variants. PLoS One. 2012;7(8):e41266. doi: 10.1371/journal.pone.0041266. Epub  2012 Aug 14. PubMed PMID: 22916104; PubMed Central PMCID: PMC3419252. | No | Not Irish based |
| O'Kelly FD, O'Kelly CM. The natural history of injecting drug use: a 25-year  longitudinal study of a cohort of injecting drug users in inner city Dublin. Ir J  Med Sci. 2012 Dec;181(4):541-8. doi: 10.1007/s11845-012-0814-9. Epub 2012 Mar 20.  PubMed PMID: 22430070. | Yes |  |
| Whitaker T, Ryan P, Cox G. Stigmatization among drug-using sex workers  accessing support services in Dublin. Qual Health Res. 2011 Aug;21(8):1086-100.  doi: 10.1177/1049732311404031. Epub 2011 Apr 6. PubMed PMID: 21471427. | Yes |  |
| Swan D, Long J, Carr O, Flanagan J, Irish H, Keating S, Keaveney M, Lambert  J, McCormick PA, McKiernan S, Moloney J, Perry N, Cullen W. Barriers to and  facilitators of hepatitis C testing, management, and treatment among current and  former injecting drug users: a qualitative exploration. AIDS Patient Care STDS.  2010 Dec;24(12):753-62. doi: 10.1089/apc.2010.0142. PubMed PMID: 21138381. | Yes |  |
| Collins R, Ewing D, Boggs B, Taggart N, Drillingcourt A, Kelly M, Patterson  D. Opiate substitution prescribing in Belfast - two year follow up study. Ir J  Psychol Med. 2009 Dec;26(4):183-186. doi: 10.1017/S0790966700000665. PubMed PMID:  30282239. | No | Not inclusive of study populaton |
| Noonan A, Kavanagh P, Sweeney B. Drug users' failure to modify alcohol  consumption in response to hepatitis C. Ir J Psychol Med. 2009 Mar;26(1):27-31.  doi: 10.1017/S0790966700000100. PubMed PMID: 30282279. | Yes |  |
| Peirce C, Coffey JC, O'Grady H, Aly S, O'Malley K, O'Donohoe M. The  management of mycotic femoral pseudoaneurysms in intravenous drug abusers. Ann  Vasc Surg. 2009 May-Jun;23(3):345-9. doi: 10.1016/j.avsg.2008.08.013. Epub 2008  Sep 21. PubMed PMID: 18809282. | No | Not specific to study populaton |
| O'Connor G, McMahon G. Complications of heroin abuse. Eur J Emerg Med. 2008  Apr;15(2):104-6. doi: 10.1097/MEJ.0b013e3282f08aa4. Review. PubMed PMID:  18446076. | No | Does not report on HCV related issues |
| Cullen W, Stanley J, Langton D, Kelly Y, Bury G. Management of hepatitis C  among drug users attending general practice in Ireland: baseline data from the  Dublin area hepatitis C in general practice initiative. Eur J Gen Pract.  2007;13(1):5-12. PubMed PMID: 17366287. | Yes |  |
| Bannan N, Rooney S, O'connor J. Zopiclone misuse: an update from Dublin. Drug  Alcohol Rev. 2007 Jan;26(1):83-5. PubMed PMID: 17364840. | No | Does not report on HCV |
| March JC, Oviedo-Joekes E, Romero M. Factors associated with reported  hepatitis C and HIV among injecting drug users in ten European cities. Enferm  Infecc Microbiol Clin. 2007 Feb;25(2):91-7. PubMed PMID: 17288906. | No | Not specific to study population |
| Danis K, Doherty L, McCartney M, McCarrol J, Kennedy H. Hepatitis and HIV in  Northern Ireland prisons: a cross-sectional study. Euro Surveill. 2007 Jan  1;12(1). pii: 674. PubMed PMID: 27938649. | No | Not specific to ROI |
| Cullen W, Stanley J, Langton D, Kelly Y, Staines A, Bury G. Hepatitis C  infection among injecting drug users in general practice: a cluster randomised  controlled trial of clinical guidelines' implementation. Br J Gen Pract. 2006  Nov;56(532):848-56. PubMed PMID: 17132352; PubMed Central PMCID: PMC1927093. | Yes |  |
| Long J, Keenan E, Grogan L, Mullen L, Barry J, Sinclair H. HIV infection  among heroin users and area of residence. Ir Med J. 2006 Sep;99(8):230-3. PubMed  PMID: 17120604. | No | Reports on HIV, not HCV |
| Hopkins S, Lambourne J, Farrell G, McCullagh L, Hennessy M, Clarke S, Mulcahy  F, Bergin C. Role of individualization of hepatitis C virus (HCV) therapy  duration in HIV/HCV-coinfected individuals. HIV Med. 2006 May;7(4):248-54. PubMed  PMID: 16630037. | Yes |  |
| March JC, Oviedo-Joekes E, Romero M. Drugs and social exclusion in ten  European cities. Eur Addict Res. 2006;12(1):33-41. PubMed PMID: 16352901. | No | Not specific to population |
| Grogan L, Tiernan M, Geogeghan N, Smyth B, Keenan E. Bloodborne virus  infections among drug users in Ireland: a retrospective cross-sectional survey of  screening, prevalence, incidence and hepatitis B immunisation uptake. Ir J Med  Sci. 2005 Apr-Jun;174(2):14-20. PubMed PMID: 16094907. | Yes |  |
| Cullen W, Kelly Y, Stanley J, Langton D, Bury G. Experience of hepatitis C  among current or former heroin users attending general practice. Ir Med J. 2005  Mar;98(3):73-4. PubMed PMID: 15869062. | Yes |  |
| Keating S, Coughlan S, Connell J, Sweeney B, Keenan E. Hepatitis C viral  clearance in an intravenous drug-using cohort in the Dublin area. Ir J Med Sci.  2005 Jan-Mar;174(1):37-41. PubMed PMID: 15868888. | Yes |  |
| Barry J, Bourke M, Buckley M, Coughlan B, Crowley D, Cullen W, Dooley S,  Keating S, Kelleher D, Moloney J, Murray F, McCormick PA, MacMathuna P, O'Connor  J, O'Grady J, O'Sullivan C, O'Sullivan P, Quinn C, Smyth B, Sweeney B; Dublin  Area Hepatitis C Initiative Group. Hepatitis C among drug users: consensus  guidelines on management in general practice. Ir J Med Sci. 2004  Jul-Sep;173(3):145-50. Review. PubMed PMID: 15693384. | Yes |  |
| Smyth BP, Barry J, Keenan E. Irish injecting drug users and hepatitis C: the  importance of the social context of injecting. Int J Epidemiol. 2005  Feb;34(1):166-72. Epub 2004 Oct 28. PubMed PMID: 15513970. | Yes |  |
| Jürgens R. Dublin Declaration on HIV/AIDS in prisons launched. Can HIV AIDS  Policy Law Rev. 2004 Apr;9(1):40. PubMed PMID: 15216826. | No | Does not report on HCV-related issues |
| Sweeney B. High morbidity expected from cirrhosis in injecting drug users. Ir  Med J. 2004 Feb;97(2):56. PubMed PMID: 15134277. | Yes |  |
| Lines R. Ireland: HIV and hepatitis C in prisons. Can HIV AIDS Policy Law  Rev. 2002 Mar;6(3):17-9. PubMed PMID: 14979231. | No | Commentary on previously included studies |
| Kavanagh P, Moloney J, Quinn C, O'Kelly E, McCormick PA. High morbidity  expected from cirrhosis in injecting drug users. Ir Med J. 2003  Nov-Dec;96(10):303-5. PubMed PMID: 14870809. | Yes |  |
| Jürgens R. HIV/AIDS in prisons: recent developments. Can HIV AIDS Policy Law  Rev. 2002 Dec;7(2-3):13-20. English, French. PubMed PMID: 14719487. | No | Does not report on HCV-related issues |
| Cullen W, Bury G, Barry J, O'Kelly FD. Hepatitis C infection among drug users  attending general practice. Ir J Med Sci. 2003 Jul-Sep;172(3):123-7. PubMed PMID:  14700114. | Yes |  |
| Balogun MA, Ramsay ME, Parry JV, Donovan L, Andrews NJ, Newham JA, McGarrigle  C, Harris KA, Teo CG. A national survey of genitourinary medicine clinic  attenders provides little evidence of sexual transmission of hepatitis C virus  infection. Sex Transm Infect. 2003 Aug;79(4):301-6. PubMed PMID: 12902580; PubMed  Central PMCID: PMC1744706. | No | Not inclusive of ROI |
| Smyth BP, O'Connor JJ, Barry J, Keenan E. Retrospective cohort study  examining incidence of HIV and hepatitis C infection among injecting drug users  in Dublin. J Epidemiol Community Health. 2003 Apr;57(4):310-1. PubMed PMID:  12646549; PubMed Central PMCID: PMC1732426. | Yes |  |
| Lines R. Irish prison guards call for expansion of methadone access. Can HIV  AIDS Policy Law Rev. 2001;6(1-2):71-4. English, French. PubMed PMID: 11837036. | No | Not specific to HCV |
| Clarke S, Keenan E, Bergin C, Lyons F, Hopkins S, Mulcahy F. The changing  epidemiology of HIV infection in injecting drug users in Dublin, Ireland. HIV  Med. 2001 Oct;2(4):236-40. PubMed PMID: 11737403. | No | Reports on HIV |
| Long J, Allwright S, Barry J, Reynolds SR, Thornton L, Bradley F, Parry JV.  Prevalence of antibodies to hepatitis B, hepatitis C, and HIV and risk factors in  entrants to Irish prisons: a national cross sectional survey. BMJ. 2001 Nov  24;323(7323):1209-13. PubMed PMID: 11719410; PubMed Central PMCID: PMC59992. | Yes |  |
| Fitzgerald M, Barry J, O'Sullivan P, Thornton L. Blood-borne infections in  Dublin's opiate users. Ir J Med Sci. 2001 Jan-Mar;170(1):32-4. PubMed PMID:  11440409. | Yes |  |
| Goulding C, O'Connell P, Murray FE. Prevalence of fibromyalgia, anxiety and  depression in chronic hepatitis C virus infection: relationship to RT-PCR status  and mode of acquisition. Eur J Gastroenterol Hepatol. 2001 May;13(5):507-11.  PubMed PMID: 11396529. | Yes |  |
| Thornton L, Barry J, Long J, Allwright S, Bradley F, Parry JV. Comparison  between self-reported hepatitis B, hepatitis C, and HIV antibody status and oral  fluid assay results in Irish prisoners. Commun Dis Public Health. 2000  Dec;3(4):253-5. PubMed PMID: 11280253. | Yes |  |
| Smyth BP. Health effects of prisons. Many injectors stop injecting while  imprisoned. BMJ. 2000 Dec 2;321(7273):1406. PubMed PMID: 11099291; PubMed Central  PMCID: PMC1119121. | Yes |  |
| Clarke SM, Mulcahy FM. Antiretroviral therapy for drug users. Int J STD AIDS.  2000 Oct;11(10):627-31. Review. PubMed PMID: 11057932. | No | Does not report on HCV |
| Smyth BP, Keenan E, O'Connor JJ. Assessment of hepatitis C infection in  injecting drug users attending an addiction treatment clinic. Ir J Med Sci. 2000  Apr-Jun;169(2):129-32. PubMed PMID: 11006671. | Yes |  |
| Allwright S, Bradley F, Long J, Barry J, Thornton L, Parry JV. Prevalence of  antibodies to hepatitis B, hepatitis C, and HIV and risk factors in Irish  prisoners: results of a national cross sectional survey. BMJ. 2000 Jul  8;321(7253):78-82. PubMed PMID: 10884256; PubMed Central PMCID: PMC27426. | Yes |  |
| Smyth BP, Keenan E, O'Connor JJ. Evaluation of the impact of Dublin's  expanded harm reduction programme on prevalence of hepatitis C among short-term  injecting drug users. J Epidemiol Community Health. 1999 Jul;53(7):434-5. PubMed  PMID: 10492738; PubMed Central PMCID: PMC1756924. | Yes |  |
| Birchard K. Inmates in Irish prisons face drug abuse and disease. Lancet.  1999 Aug 28;354(9180):753. PubMed PMID: 10475200. | No | Does not report on HCV related issues |
| Smyth BP, Keenan E, O'Connor JJ. Bloodborne viral infection in Irish  injecting drug users. Addiction. 1998 Nov;93(11):1649-56. PubMed PMID: 9926528. | Yes |  |
| McDonnell RJ, McDonnell PM, O'Neill M, Mulcahy F. Health risk profile of  prostitutes in Dublin. Int J STD AIDS. 1998 Aug;9(8):485-8. PubMed PMID: 9702600. | No | Not specific to study population |
| Smyth R, Keenan E, Dorman A, O'Connor J. Hepatitis C infection among  injecting drug users attending the National Drug Treatment Centre. Ir J Med Sci.  1995 Oct-Dec;164(4):267-8. PubMed PMID: 8522425. | Yes |  |
